# Evaluation of Oxford Nanopore’s MinION Sequencing Device for Microbial Whole Genome Sequencing Applications

**DOI:** 10.1038/s41598-018-29334-5

**Published:** 2018-07-19

**Authors:** Andrea D. Tyler, Laura Mataseje, Chantel J. Urfano, Lisa Schmidt, Kym S. Antonation, Michael R. Mulvey, Cindi R. Corbett

**Affiliations:** 1 National Microbiology Laboratory, Public Health Agency of Canada, 1015 Arlington Street, Winnipeg, Manitoba, R3E 3R2 Canada; 2 University of Manitoba, Department of Medical Microbiology and Infectious Diseases, Winnipeg, Manitoba, R3E 0J9 Canada

## Abstract

The MinION sequencer (Oxford Nanopore Technologies) is a paradigm shifting device allowing rapid, real time long read sequencing of nucleic acids. Yet external benchmarking of this technologies’ capabilities has not been extensively reported, nor has thorough evaluation of its utility for field-based analysis with sub-optimal sample types been described. The aim of this study was to evaluate the capability of the MinION sequencer for bacterial genomic and metagenomic applications, with specific emphasis placed on the quality, yield, and accuracy of generated sequence data. Two independent laboratories at the National Microbiology Laboratory (Public Health Agency of Canada), sequenced a set of microbes in replicate, using the currently available flowcells, sequencing chemistries, and software available at the time of the experiment. Overall sequencing yield and quality improved through the course of this set of experiments. Sequencing alignment accuracy was high reaching 97% for all 2D experiments, though was slightly lower for 1D sequencing (94%). 1D sequencing provided much longer sequences than 2D. Both sequencing chemistries performed equally well in constructing genomic assemblies. There was evidence of barcode cross-over using both the native and PCR barcoding methods. Despite the sub-optimal nature of samples sequenced in the field, sequences attributable to *B. anthracis* the target organism used in this scenario, could none-the-less be detected. Together, this report showcases the rapid advancement in this technology and its utility in the context of genomic sequencing of microbial isolates of importance to public health.

## Introduction

Oxford Nanopore Technonologies’ (ONT) MinION is a pocket-sized device which applies nanopore sequencing technology to nucleic acid analyses, with far reaching applications including real-time bacterial metagenomic community analysis, subtyping, and long read scaffolding for whole genome sequencing of organisms, to name but a few^1^. Although this technology continues to evolve, the MinION’s small, agile physical presence, and long-read capabilities have led to its use in several important microbiological studies including studies of Ebola, Zika virus and for the detection of vancomycin resistant Enterococci^2–5^. In the context of biothreat agent identification in the field for triage of unknown samples, this capability holds promise as it has the potential to perform unbiased identification and characterization of these agents, as well as to aid in source attribution of samples through capture of other extraneously present DNA.

Despite the potentially paradigm shifting nature of this sequencing approach, the speed of evolution of both the experimental chemistry and data analysis has made external benchmarking or operational validation of the MinION device in a remote or laboratory setting troublesome. Furthermore, characterization of the limitations of the technology in terms of broad error classes, biases and inter run variability have not been widely described. While large consortia such as the MinION analysis and reference consortium (MARC) also strive to document sequencing capability, the results which have been presented to date have yet to be updated along with the technology, and have described results in a narrow set of samples^6,7^. Furthermore, there has been no extensive external evaluation of its metagenomic detection capability in well characterized or sub-optimal samples.

In order to document the quality of data currently being generated by ONT’s MinION device using R9.4 flowcells with the 2D, 1D and rapid 1D chemistry, two independent laboratory groups within our institution were tasked with sequencing a set of well characterized samples, and comparing the data generated with that collected from other sequencing methods, as well as NCBI references as available. At the time of writing this manuscript, 2D sequencing in which both strands of the DNA molecule are sequenced sequentially, via ligation of the strands with a hairpin adapter, represented the method of choice for generating high quality data. Both 1D and rapid 1D sequencing involve evaluation of a single strand of each DNA molecule, thus generating data of slightly lower quality, although at higher sequencing efficiency. The aim of this analysis was to independently assess the sequencing capability of the MinION device as it has matured over time, addressing measurements of accuracy, reproducibility, *de novo* assembly contiguity, and sequencing bias in both idealized samples, as well as in lower quality samples typical of a field-based scenario. We also describe utilization of the ONT MinION in a national biological security exercise to assess its capability for rapid, on site investigation of exhibits contaminated with biothreat agents in the field. The National Microbiology Laboratory (NML, Winnipeg, Canada), is a world class institution in both reference and diagnostic testing and protocol development for assays involving microbial organisms of importance for public health and bioforensic analyses. It is also the operational centre of Canada’s Microbiological Emergency Response Team for public health security. For each of these mandates, accurate and realistic measures of new technologies’ advantages and limitations are important. Thus, evaluating the sequencing capabilities of the MinION is an important step forward in ensuring accuracy of clinical and research observations both in a laboratory and remote setting.

## Results

15 Runs were carried out over a nine month period from 2016–2017. The experiments tested 2D, 2D sequencing with barcoding, 1D sequencing with barcoding and 1D rapid kits, and applied the most up-to-date software version at the time of data generation for analysis (Supplementary Table 1).

### Flowcell Quality - Porecounts

All R9.4 and R9.4 spotON flowcells were received with porecounts exceeding the guaranteed level (800 pores). Porecounts at the time of use were measured, with results depicted in Supplementary Table 2. Early R9.4 flowcells, (non-SpotON) that were stored for longer periods prior to use (4 °C as per ONT), were for the most part, of lower quality at the time of sequencing than immediately upon receipt. Later flowcells (R9.4 SpotON) were of equal, or even higher quality after storage likely due to changes in pore evaluation protocols implemented by the MinKNOW software.

### Sequencing Yield

**Figure 1:**
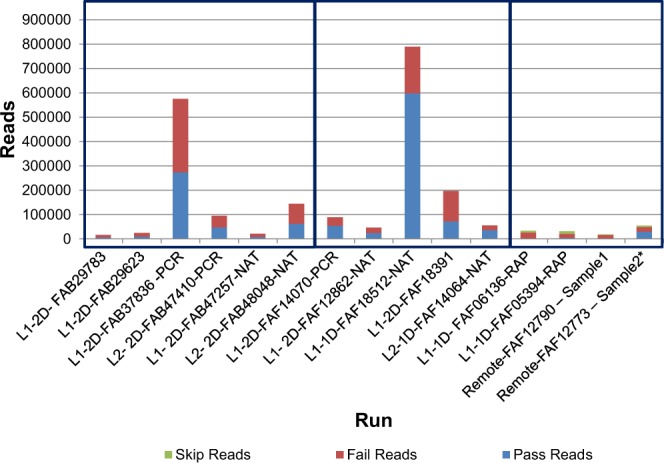
DNA yield in number of reads which were analyzed further (pass) and reads which were excluded based on either internal Epi2Me filtering parameters or inability to demultiplex (Fail), on each of the flowcells tested. FAB-R9.4; FAF- R9.4 SpotOn. The first set of samples were assessed using Epi2Me, the second set by Albacore, and the third by MinKNOW direct basecalling. *This sample had a second top up run added 1 hour following the commencement of sequencing.

**Figure 2:**
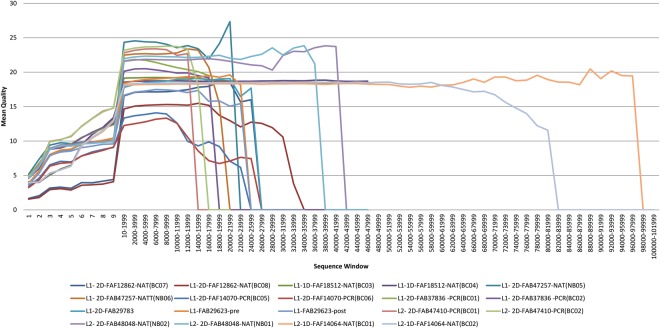
Mean quality per 2000 base pair window across each of the MinION runs included in this analysis calculated using fastqc^32^. Lines extend further as the length of the read increases. Earlier runs were characterized by both shorter reads, and lower quality output data generated. L1-2D-FAB29623 is broken into pre and post, referring to read quality before and after the wash buffer was applied.

Sequencing yield per run is described in Fig. 1, with both total number of reads and usable (pass) reads denoted. The first set of samples were classified as either pass or fail by Epi2Me (Metrichor’s cloud-based basecalling software) with sequences which were successfully 2D analyzed, demultiplexed, and a minimum mean quality score of 6 applied for this classification. Runs with reads basecalled by albacore are included in the second box, and included all reads which had successful 2D basecalling and were demultiplexed, regardless of the final quality score. For the final 4 runs, basecalling and demultiplexing was carried out by MinKNOW (1D). Additionally, skipped reads (those which were not processed by MinKNOW at the time of sequencing) were also generated and are also described. An average of 61,848 (maximum 273,102), 160,132 (maximum 598,238) and 9641 (maximum 29,632) passed reads were generated for the 2D, 1D and rapid 1D kits respectively. Note that both L1-2D-FAB37836-PCR and L1-1D-FAF18512-NAT runs generated substantially more data than was generated in any of the other runs. This quantity of data was not duplicated in replicate 2D or 1D runs. In our experience, as well as that described by others in Oxford Nanopore’s user community, the majority of sequence data is generated in the first eight hours of sequencing, corresponding to the time in which the first group of pores is actively sequencing. Importantly, the lack of automatic filtering of output data from Albacore in later runs partially accounted for the increased proportion of reads called “Pass”. This did, however, allow us to evaluate the quality of both raw data as well as the effects of various quality control metrics on downstream analyses. Of the usable reads that were generated, there was a general increase in read quality as the study progressed, with earlier runs characterized by both shorter reads, and lower quality output data (Fig. 2).

### Effect of Sequential addition of samples on data generation

To address the effect of “stacking,” adding a secondary sample to a MinION flowcell, either following a wash step or by simply adding it to an ongoing run, Laboratory 1 (L1) sequenced *F. hispaniensis* and *Y. rohdei* sequentially. When *F. hispaniensis* was sequenced first, followed by stopping the sequencing software, applying wash buffer and restarting the run, we observed a drastic drop in actively sequencing pores following the restart (1092 to 555). For the four hours prior to the wash, 9003 2D pass reads were generated. 99.8% of these were successfully mapped to *F. hispaniensis* corresponding to a calculated average coverage of 35.9x. This high rate of purity is expected given that this was the only organism deposited on the flowcell at this time. During the subsequent 48 hours in which the flowcell ran following application of the wash buffer and addition of *Y. rohdei*, only 751 successfully 2D called reads were generated. Of these 751 reads, 31.2% corresponded to the *F. hispaniensis* genome, while 67% mapped to *Y. rohdei*, with neither achieving genomic coverage exceeding 1x.

In contrast, when we conducted a similar analysis without the application of an intermediary wash step, no pores were lost following addition of the secondary sample (also added at four hours). Data was collected for eight hours in total, in two runs in which the smaller *F. hispaniensis* was sequenced first, followed by the larger *Y. rohdei*, and vice-versa. The first run generated 6038 pass 2D reads, while the second generated 71,618. Despite the wide discrepancy in the number of reads generated, the relative proportion of sequencing data comprised of the organism added after four hours of sequencing was similarly low. In the first run, in which *F. hispaniensis* was sequenced first, only 5.2% of reads overall corresponded to the *Y. rohdei* genome. In the latter run in which *Y. rodhei* was sequenced first, reads corresponding to this organism made up 91.1% of the overall reads, whereas *F. hispaniensis* reads made up only 0.4%. Interestingly in this run, over 8% of reads were unmapped. Many of these unmapped reads may have corresponded to the *F. hispaniensis* pFSC454 plasmid, which was not available for comparison in Refseq at the time of this analysis.

### Fidelity of native and PCR barcodes in 2D and 1D experiments

For 2D-PCR runs, an average of 8.4% of reads were not successfully demultiplexed based on their barcode sequence, with an additional 0.1% of these reads classified as belonging to barcodes which were not included in the experiment. In the 2D-native runs, the number of unclassified reads was lower than that observed in the PCR multiplexed runs for the initial experiment (3.3%), but increased to 43% in the later run, suggesting that the updated demultiplexing algorithm applied by the Albacore software resulted in a larger number of sequences being excluded due to alterations in the barcode detection algorithm. In all runs, the reads which were incorrectly classified as belonging to an unused barcode were negligible (0.02% and 0 respectively). This increased stringency was also observed in the analysis of the 1D native run, where 35% of reads were unclassified, and only 23 (<0.01%) were attributed to a non-existent barcode.

The majority of reads which were successfully demultiplexed could be mapped to the correct reference genome. However, there was on average, an additional 0.3% of reads in the 2D PCR barcoded runs, 0.8% for the 2D native barcode runs, and 1% in the 1D native barcoded runs which mapped to the genome of the organism with which they were co-sequenced.

### Mapping-based alignment for determination of coverage statistics and regions of bias

**Figure 3:**
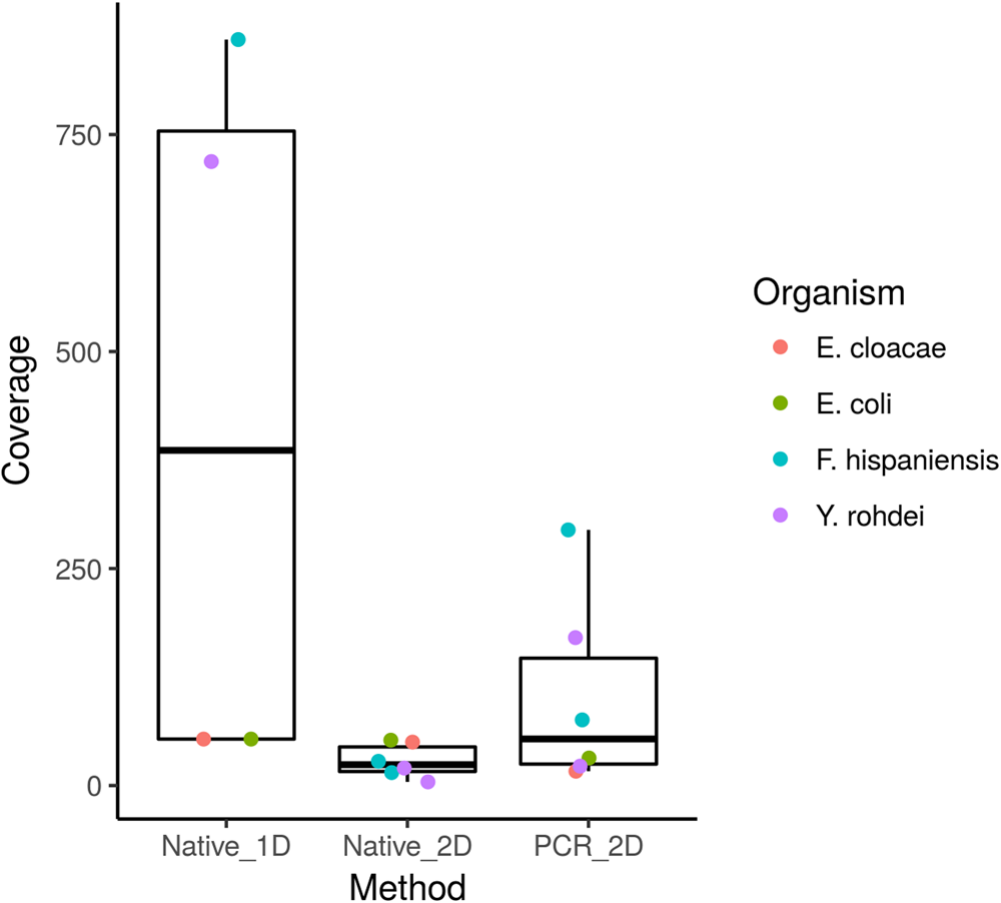
Boxplot depicting the mean genomic coverage obtained across each of the reference genomes included in this analysis. Sequenced organisms and the corresponding average levels of coverage for each are coloured as described.

Coverage across the reference genome was directly related to the number of passed reads obtained from a sequencing run (Fig. 3). We did not identify specific genomic factors which were associated with either increased or decreased coverage and therefore suggestive of sequencing bias, although interestingly the data from Laboratory 2 (L2) suggested that the isolate genomes were preferentially sequenced, at approximately a 2:1 coverage ratio when compared with any of the plasmids.

**Figure 4:**
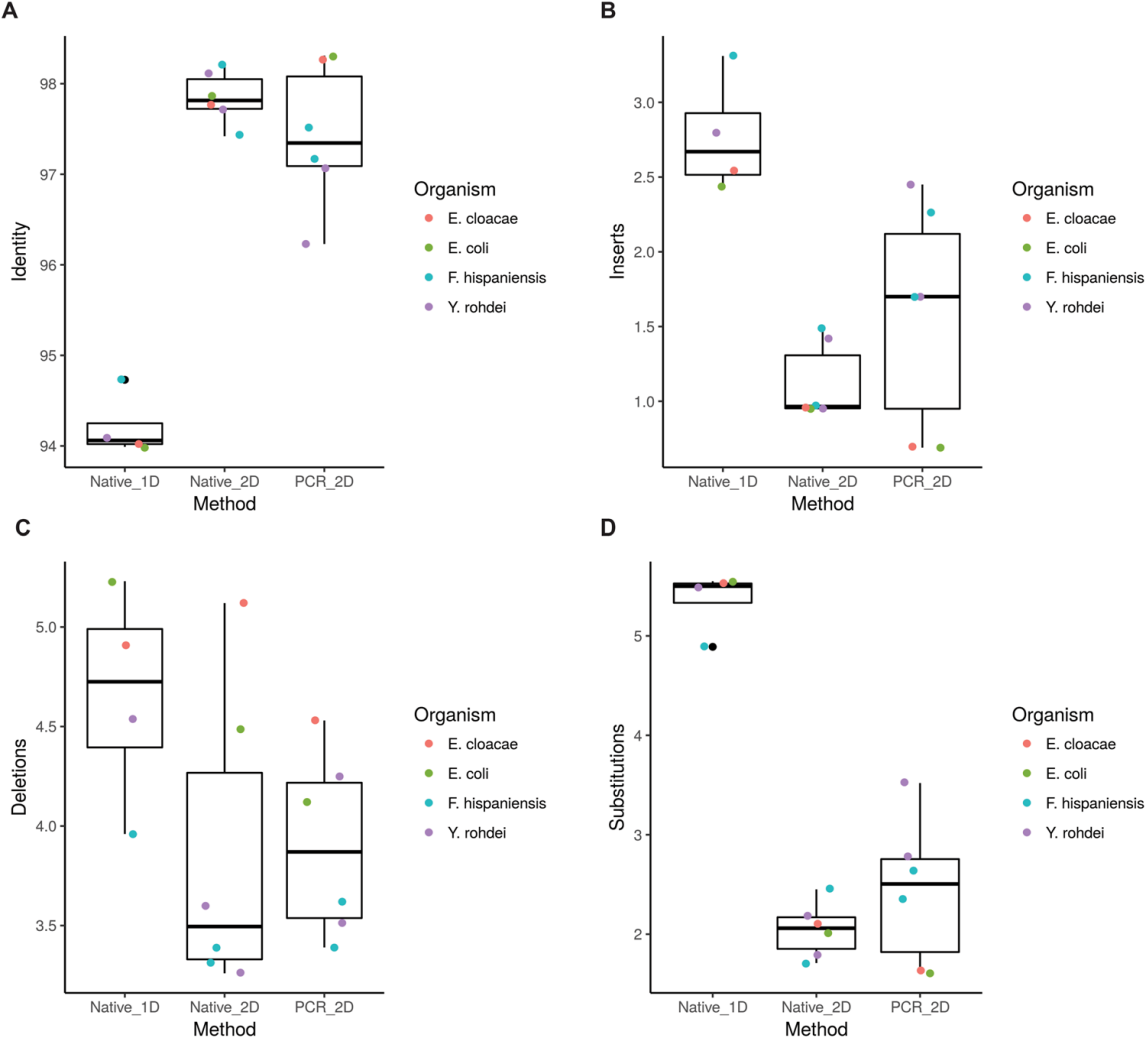
Error characteristics of data generated in various sequencing runs included in this analysis. (**A**) Proportion of bases accurately matching mapped segment of the reference. (**B**) Insertions per 100 aligned bases. (**C**) Deletions per 100 aligned bases. (**D**) Substitutions per 100 aligned bases.

Through comparison of the different sequencing methods (1D native, 2D PCR/native barcoding), we determined that the error profiles of the sequencing data generated were similar between methods, with a slight increase in error with the PCR method, and an increased error rate with 1D sequencing (Fig. 4). 2D sequencing accuracy was high, with aligned base identity approaching or exceeding 97% for all 2D experiments. The alignment accuracy for 1D sequencing while lower than that of 2D experiments, was nonetheless above 94%. 1D insertion, deletion and substitution rates were all higher than in either of the 2D barcoding experiments performed. Indel sizes ranged in average length from 1.5–1.85 base pairs (bp), suggesting that the majority of erroneous base calls were the result of skipped or added single bases. In L2′s data, errors were predominantly driven by indels associated with homopolymeric stretches (Supplementary Fig. 1). The same observation was not made in data generated by L1, suggesting that the quality of the reference used for analysis is important in interpreting error rate calculations: While L1 samples had high quality complete genomes used as reference from NCBI, references from L2 were comprised of the fasta file generated from the *de novo* assembly and polishing of MinION data with high depth MiSeq data.

### *De novo* assembly of MinION sequencing reads and cumulative accuracy of consensus contigs

*De novo* assemblies were successfully generated from both 1D and 2D data sets using Canu. When mean coverage of greater than 20 was obtained for the organisms sequenced by L1, the correct number of contigs were identified in all samples. Namely, a single large contig aligning with the *Y. rohdei* chromosome was detected in *Y. rohdei* samples, and two contigs including a chromosome and plasmid matching sequence were detected in *F. hispaniensis*. Assembly contig sizes were also approximately comparable to reference sizes. However, for the first native barcoding 2D run performed by L1, the number of reads generated did not meet the 20x threshold recommended for use with Canu. In this case, a large contig that was shorter than the expected length of the *F. hispaniensis* chromosome, and two small additional contigs were generated. For the larger, *Y. rohdei*, the genome could not be generated at all, with the largest contig created only 164 kbp in length. For the PCR barcoded run from L2, neither of the included organisms were sequenced to a sufficient depth to allow for construction of a complete set of contigs. In this run, the largest contig generated for the *E. coli* sample was 839 kbp, and was 62 kbp for *E. cloacae*. Sufficient depth was achieved when sequencing using both the 1D and 2D native chemistries to generate appropriate genomic scaffolds for each of the organisms, with *E. cloacae* having a chromosome approximately 4.6 Mbp long with a single plasmid, and *E. coli* 4.7 Mbp, with a set of three additional plasmid sequences.

### Sequencing depth requirements for 1D datasets

**Figure 5:**
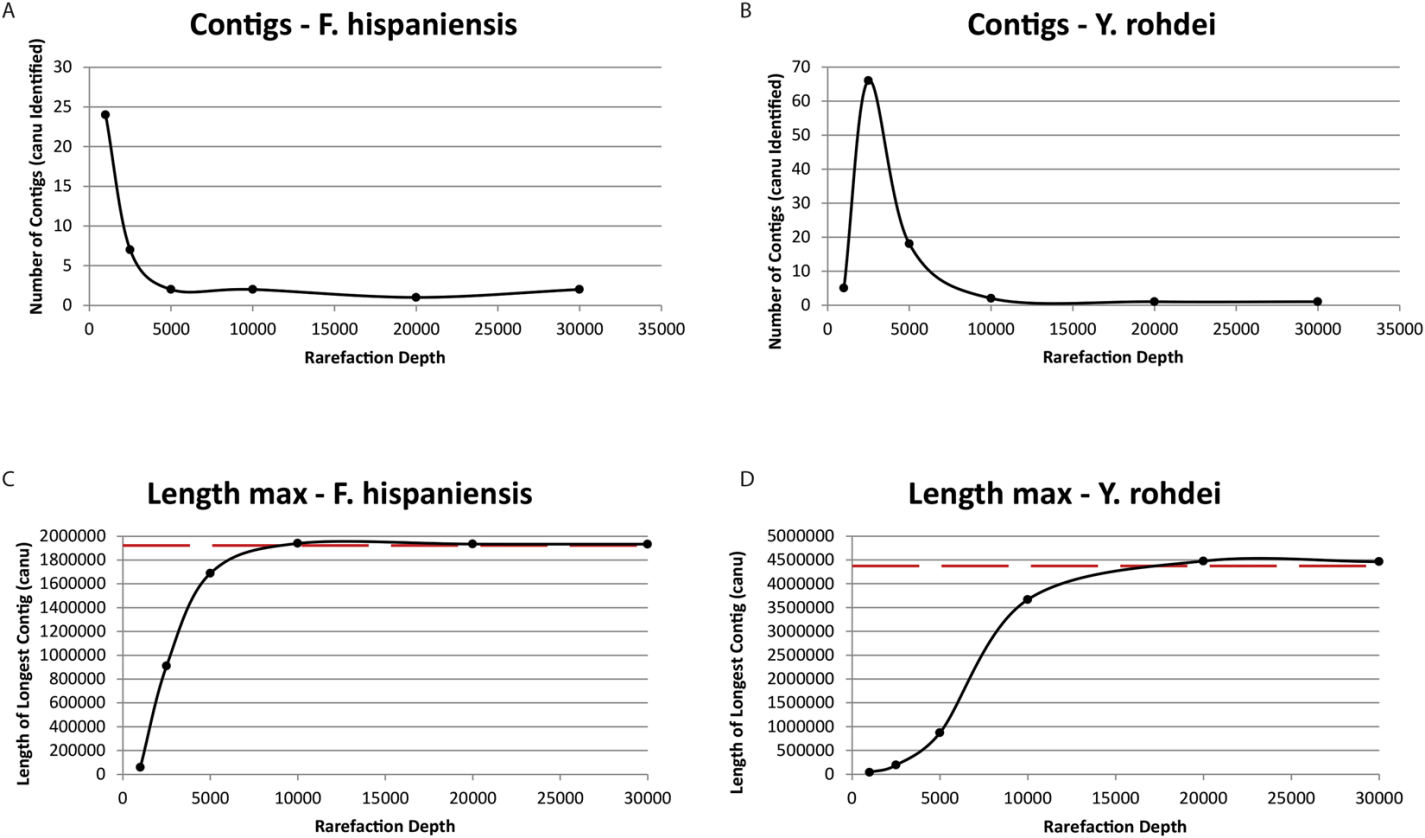
Assembly characteristics of contigs generated following subsampling of fastq reads generated with 1D sequencing and native barcoding. Dashed line is the reference genome size reported by NCBI.

In order to evaluate the required quantity of data necessary to generate accurate *de novo* assemblies, while at the same time maximizing the number of samples concurrently sequenced, specifically when using the 1D chemistry, sequences from the L1-1D-FAF18512-NAT barcoding run were rarefied at different depths of sequencing with assembly error rates and alignment accuracy evaluated at each iteration. For the smaller genome (~1.9Mbp), *F. hispaniensis*, 10,000 (10k) passed reads were theoretically sufficient to produce a single contig approximating the size of the genome, whereas for *Y. rohdei*, 20 k reads classified as pass were required to similarly generate the appropriate number and sized contigs (Fig. 5). Interestingly, the *F. hispaniensis* pFSC454 plasmid was not confidently detected in the 20k read rarefaction, suggesting that our random read selection may have dropped reads from this structure by chance, or that assembly parameters must be adjusted in the context of high coverage runs for plasmid detection. When comparing the accuracy of these assemblies in terms of SNPs detected and breakpoints required to satisfy alignments with references, contigs retained errors even when 30k pass reads were used. For the *F. hispaniensis* assemblies the number of substitutions and indels detected decreased from the 10k read iteration to the 30k read iteration (173-99 substitutions and 9357-6988 indels, respectively). Importantly, however, even with the full dataset, substitution and indel errors were detected when compared to the NCBI reference. There were also consistently six breakpoints across these iterations, suggesting larger scale genomic rearrangements characterized the assemblies generated, and that these could not be mitigated with increased read depth. For the larger *Y. rohdei* genome, approximately double the number of breakpoints were detected in the 10k iteration compared to either 20k or 30k (420 vs 262 and 266, respectively). There were also large scale insertions. Substitutions were consistent at approximately 18k per assembly and indels decreased from 43k in the 10k assembly to just over 24k in the 30k assembly (Supplementary Table 3). When error correction using Pilon^8^ with MiSeq Nextera XT was applied, SNP errors decreased, however the number of breakpoints (representative of mis-assemblies) did not improve for either organism. FLYE, an Abruijn method^9^ was evaluated to determine whether it might offer benefit over Canu on this type of dataset. While considerably faster, use of this method did not impact assembly quality.

### Effect of quality trimming parameters on 1D data quality

Unlike Metrichor/Epi2me and MinKNOW, reads basecalled with Albacore are not automatically classified as either pass or fail as part of the base calling process. Thus, in order to evaluate optimum trimming conditions for *de novo* assembly for a 1D MinION barcoded run, the effect of trimming reads based on different characteristics was evaluated using the full dataset from L1-1D-FAF18512-NAT. Effect of adapter contamination on assembly and read data were evaluated by trimming read ends at various levels, or applying Porechop^7^. Conservatively trimming reads by 100, or less conservatively by 50 basepairs on either end or applying Porechop did not have a notable effect on read error rate or assembly quality in terms of contigs generated or variants detected. Next evaluated was the effect of applying various length and quality cutoffs, with each of the parameters tested resulting in exclusion of different quantities of data. Over 10k (3%) reads in the *Y. rohdei* isolate were excluded when a minimum length of 1000 bases was applied. For the *F. hispaniensis* samples, approximately 14k (7%) reads were removed. Mean read quality thresholds of 10 and 20 were assessed as well, with 58 and 77 sequences removed for *Y. rohdei* and *F. hispaniensis*, respectively at a quality threshold of 10, and 286,368 (72%) and 126,804 (62%) at mean quality of 20. Co-filtering for quality >20 and length greater than 1000 bases was also performed with 287,936 (73%) and 126,804 (63%) filtered from the *Y. rohdei* and *F. hispaniensis* samples.

Interestingly there was little difference between the sequencing data quality in terms of introduced alignment errors between any of these filtering methods. Assembly accuracy and coverage (Supplementary Table 3) across the contig was also similar between all methods, likely due to the substantial internal screening performed by Canu prior to contig generation. Use of nanopolish^10^ reduced the frequency of indel errors, but was less successful in reducing larger rearrangements and inversions. For the *F. hispaniensis* data, all methods generated a genomic contig of approximately 1.95M basepairs concordant with the reference data. All also generated a second contig attributable to the pFSC454 plasmid. The assemblies of this plasmid generated by each of the methods were longer than the 16k basepairs reported by NCBI (NZ_CP018094; April 16, 2017), at ~110k basepairs for the trimmed end filtering 63k basepairs when minimum quality was set to 20 (including the iteration when quality and length were filtered), and 38k basepairs for the rest of the analyses. For the *Y. rohdei* analysis, each of the filtering methods generated a large contig of 4.5M basepairs. Interestingly, for the iterations in which a quality filtering threshold of 20 and length greater than 1000 was used, four additional contigs with lengths ranging from 17k basepairs to 40k basepairs were also generated. A BLAST search identified these as part of the *F. hispaniensis* genome, suggesting that in samples which are substantially filtered, secondary contaminant genomes from other concurrently run samples, especially those with small genomes, may make up a substantial enough proportion of reads to be assembled. Coverage across these contigs was much lower than that across the intentionally sequenced genome in question (5x), suggesting that application of a contig coverage threshold could mitigate reporting of contaminant assemblies, when a sufficient number of reads are generated in a run.

Misassemblies identified by breakpoints and inversions in contig comparison with the reference, were detected across all iterations, and did not improve substantially upon application of more robust screening procedures or with the use of nanopolish (Supplementary Table 3). No filtering method successfully removed all errors from the contigs, suggesting either that despite the high degree of coverage achieved in this experiment that it was still ineffective in accurately assembling the genomes, or that there are errors in the NCBI Refseq entries for these organisms.

### Pre-deployment assessment of the performance of the MinION 1D rapid kit

The majority of reads generated by rapid 1D sequencing were produced in the first 16 hours of sequencing. However, most were classified by MinKNOW as fail or were skipped (basecalling not performed) (Fig. 1). The median quality for MinKNOW passed reads for the *F. hispaniensis* run was 10. Of the reads which were skipped by MinKNOW in the *F. hispaniensis* run, 5839 of 7532 were successfully basecalled using albacore, with fastq data extracted using poretools. However, these were of lower median quality (5.5, poretools default settings) than were sequencing reads which were classified as pass by the MinKNOW device. When length-based filtering was turned off, the median quality of skipped reads was 6. Basecalling of reads skipped by MinKNOW also added substantially to the turn-around time of the assay, with this step requiring an additional 15 hours of processing time. For this reason, it was decided that subsequent experiments would focus on only the base called data which was classified as pass by MinKNOW. Median quality for the pass reads of the *Y.rohdei* run was 9. Notably, freshly prepared DNA was not used for this set of experiments, potentially explaining the reduced quantity of generated reads.

Sequencing reads classified as pass in this experiment were subsequently compared against the *F. hispaniensis* (NZ CP018093.1) and *Y.rohdei* (NZ CP009787.1) genomes, using NanoOK (Table 1). Read quality and aligned base identity were lower than that previously described for 1D data. In order to evaluate our ability to correctly identify pathogens, despite this decrease in quality, we ran sequenced fastqs through kraken on a desktop computer with 48 2.30 GHz cores. The 1277 sequences from the *F. hispaniensis* run were processed in 1241.935 s. 1077 (84.34%) were classified correctly, and 200 (15.66%) were unclassified, erroneous or not assigned below the family level. For the *Y.rohdei* analysis, 5044 sequences were processed in 1680.814 s. Of these, 4279 (84.83%) were classified and 765 (15.17%) were unclassified, erroneous or not assigned below the family level. We also evaluated the effect of read quality filtering on the passed reads, applying a mean q-score cutoff of 8, to determine whether this had an effect on the ability to classify reads to the appropriate taxonomic group. When we applied this filter to the *F. hispaniensis* data, 1043 sequences surpassed this threshold, of which 97.03% were classified correctly at the genus level. For *Y. rohdei* 4105 sequences passed this threshold with 94.91% classified appropriately (genus). The results of these experiments were used to inform decision making in analyses conducted outside of the lab.

**Table 1:** Sequence quality of data generated using the MinION sequencing device with the 1D rapid kit from ONT (SQK-RAD002).

	Number of Reads	Reads with alignment	Aligned base identity	Identical Bases per 100 aligned	Inserted based per 100 aligned	Deleted bases per 100 aligned	Substitutions per 100 aligned
L1-1D- FAF06136-RAP - Pass	1277	1218	89.04%	79.50%	2.91%	7.80%	9.79%
L1-1D-FAF05394-RAP -Pass	5044	4731	92.04%	82.83%	1.53%	8.47%	7.16%

Data shown were obtained from a pair of isolates run in the laboratory, and used for benchmarking the technologies’ capabilities using NanoOK. *F. hispaniensis* = *Francisella hispaniensis*; *Y. rohdei* = *Yersinia rohdei*.

### Sequencing metrics of data generated remotely

Sample one which was composed of “pure” *B. anthracis* Vollum strain genomic DNA (1.3 ng/uL; 6.5 ng sequenced), was sequenced for a total of five hours and generated much less data than that typical of sequencing runs carried out under ideal experimental conditions (Table 2). Sample two, a swab in TE, in which 5 uL containing a mix of *B. anthracis* (3.3 ng) and control human genomic DNA (5ug), produced much more data (Table 2). For the sequencing of Sample two, the addition of a second library preparation to the same flowcell one hour after sequencing began, resulted in generation of a comparable amount of sequence data to output obtained from non-rapid kit testing after 23 hours of run time. It is noteworthy that for these experiments DNA concentrations well below the 27 ng/uL recommended by ONT were used. Despite this challenge, for both runs, sufficient sequence data was available after approximately one hour, to allow analysis.

**Table 2:** Primary sequencing output metrics characterizing data obtained from two MinION runs carried out at a remote Canadian location.

	L1-1D- FAF06136-RAP	L1-1D-FAF05394-RAP	Sample 1 T1	Sample 1 T-final	Sample 2 T1**	Sample 2 T-final
Elapsed time	26 h	26 h	49 min	5 hr	60 min	23 hr
Total sequences generated	33467	31462	3389	14855	3292	54619
Pass (minKNOW) sequences	1278	5044	472	2611	1328	29623
Reads skipped by local basecaller	7532	11140	219	2044	249	5866
Pass (mean q > 8) sequences	1043	4105	365	1994	1013	2971

^**^Second library prepared from the same sample was added at this time.

### Pathogen identification and biological triage capability

In order to fully evaluate the biological triage capability of the MinION, several tools were used to taxonomically assign sequencing reads to increase confidence in identification. Sample one at T1 had 78.4% of reads assigned to the *B. cereus* group by kraken, with the 20.27% classified as *B. anthracis* and an additional 8% assigned to *B. cereus* or *B. thuringensis*. At the genus level, 88.5% of reads were classified as Bacillus, with the remainder unclassified. These proportions were carried through to the final analysis (T-final) in this sample. Signature sequence analysis at T1 showed two sequences mapping to a genomic signature sequence, and 103 mapping to pXO1 or pXO2. At T-final, this sample had 18 sequences mapping to four genomic signature sequences, and an additional 418 mapping to pXO1 and pXO2. Analysis of data with MASH^11^ (T1 and T-final) provided top hits corresponding to the pXO1 plasmid of *B. anthracis* and the *B. anthracis* genome. Taken together, this data shows that we are able to accurately identify *B. anthracis* in a pure, low concentration sample, one hour after starting sequencing using the MinION technology, a time point equivalent to traditional real time PCR.

At T1, sample two had approximately 83% of the sequences classified. The vast majority (81.2%) corresponded to *Homo sapiens*. At this time, only two reads were successfully called as *B. anthracis*, while 12 (1.1%) were identified as *Escherichia coli*. Given that this sample appeared to represent a mixed community, MASH was not used for analysis. Signature sequence analysis at T1 identified no *B. anthracis* mapping reads at our cutoff level of mapping quality score (50), however, when no threshold was used, one read mapping to a genomic signature, and one to the pXO1 plasmid were detected. As such, additional library was prepared and added to the flowcell to determine whether this sample was indeed positive for *B. anthracis*. After allowing an additional 23 hours to pass, 14.5% of sequences were unclassified, and the majority classified as *H. sapiens* (83.94%). Thirteen reads were identified as members of the *B. cereus* group, with only three corresponding to *B. anthracis*. The remainder of reads were classified as *E. coli* (27; 0.47%). Signature sequence analysis identified three reads which mapped with high confidence (score >50) to pXO1 or pXO2. When no quality threshold was applied, 168 reads were successfully mapped, with the majority 98.2% mapping to either plasmid.

## Discussion

The MinION device developed by ONT has several purported advantages over traditional next generation sequencing platforms, including direct sequencing of nucleic acids allowing DNA modification detection and direct RNA sequencing to be performed, real time data acquisition and analysis, and the ability to generate long reads^1,12–17^. However, the novelty of this approach and the dearth of independent research benchmarking the technologies’ capabilities as it evolves for general use applications, is a fundamental knowledge gap which needs to be addressed. Two studies performed by the MARC evaluated the sequencing capabilities of an older version of the flowcell (R7 pores), and later the updated R9 cells with 2D and rapid 1D kits, for sequencing^6,7^. The drastic change in sequencing capability brought on by the development of new R9.4 pores, has led to the need for additional studies with evaluation carried out across a number of organisms. The present study describes the experience of two independent laboratory sections operating out of a single institution, in running ONT’s MinION device, with the updated R9.4 sequencing flowcells and corresponding 2D, 1D and 1D rapid chemistries for the analysis of a set of four well characterized isolates.

While consistent protocols between replicate runs are ideal for experimental design, in the context of the dynamic and rapid evolution of MinION-based sequencing, we were unable to maintain technical consistency between methods across all experiments. Importantly, and emblematic of this evolution, since the conclusion of this study, 2D sequencing has been replaced by 1D^2^ chemistry, in which forward and reverse strands of the DNA duplex are sequenced without being physically connected, and the rapid kit has been updated (RAD002 to RAD004). Sequencing via the 1D^2^ method has been shown to generate data of similar quality to 2D, with a greatly simplified experimental protocol, and subsequent increase in the number of reads produced^17^. This method requires updated (R9.5) flowcells and corresponding chemistries, and will need to be analyzed in a similar manner in order to assess its utility. The RAD004 kit has also been described to offer improvements in sequencing yield compared to older kit versions.

Overall the sequencing capability of the MinION device was seen to improve greatly throughout the course of our experiments, with greater DNA sequence yields generated in later runs. Changes, in particular to the MinION software, and adoption of the technically simpler 1D protocols appeared to be most responsible for the increases in yield characterizing later runs. There were, however, in both labs, dramatic inconsistencies between runs, with either lab generating significantly higher or lower yields when compared to the mean. These runs skewed results and made estimation of expected yield per flow cell, based strictly on number of samples and input DNA, challenging. The rapid kits appeared to generate somewhat similar quantities of data between runs, although the second field run, in which an additional library preparation was generated and added to the flow cell after 1 hour of sequencing, resulted in a greater amount of data produced, a procedure which, along with the use of low input kits, may be of benefit for low concentration samples.

One factor which may have an impact on output data is the quality of the input library. In our experience, 2D sequencing library generation was challenging using the protocols and reagents recommended and supplied by ONT. Evaluation of the DNA quantity at different stages of the protocol suggest that for the 2D analyses, the hairpin tagmentation step, and subsequent bead purification (final step), resulted in loss of a great deal of DNA. Consequently, several runs had to be performed with less than the recommended amount of DNA library loaded onto the flowcell. The move from 2D to 1D and rapid 1D in order to mitigate this challenge had a beneficial impact on maximizing sequencing output between runs. 1D^2^ will likely have similar effect, while at the same time retaining the high quality of data generated by sequencing both strands.

Despite the simplification of protocols characterizing rapid sequencing kits, rapid protocols still require high concentration and quality DNA as well as use of specialized laboratory equipment and technically competent staff to support the precision required for successful utilization. To address the requirement of high concentration DNA, various protocol modifications which implement either whole genome amplification (WGA) or DNA concentration techniques have been described^18,19^, and used by our group in conjunction with other sequencing methods (ie. MiSeq). Additionally, newer low input MinION library preparation kits are also available. However, in our hands, these approaches significantly increase experiment time and complexity (data not shown), rendering them impractical for rapid operational analyses. In order to evaluate the capability of the MinION in a ‘real world’ scenario in which low quantity and quality DNA is all that is available, we chose to evaluate the sequencing capability of the technology using raw extracted DNA as input to the rapid 1D sequencing kit without additional steps which may have greater utility in a laboratory setting. Low sequencing yield characterized the Y. rohdei and F. hispaniensis runs conducted in the lab, an observation which may have reflected the presence of contaminants in the DNA preparation, or a slightly longer time between extraction and sequencing. Importantly, our field results demonstrated the ability of the MinION sequencer to detect high consequence pathogen DNA even from samples in which DNA concentrations were well below those recommended by ONT for input into the rapid sequencing kits.

Our findings of sequencing accuracy above 96% across all 2D runs and above 94% for those conducted using the 1D chemistry, show promise for the utility of the technology across many disciplines. Despite progress to date, there continue to be issues with accurately calling bases in homopolymeric sequences, often leading to apparent insertions or deletions of one or a few bases^20^. This limitation has been previously described in other technologies as well, with homopolymers representing a substantial and well known challenge for accurate sequencing^21^. Indeed it is worth consideration that current reference collections also suffer from miscalls resulting from these challenging sequences^22^. Interestingly, homopolymeric stretches appeared to be more consistently miscalled in the L2 dataset. This discrepancy may be organism specific or the byproduct of different reference types employed by either lab in the analysis. This observation is of particular interest as evaluating data quality in cases in which a high quality reference genome is not available.

Generation of consensus sequences and *de novo* genome assemblies using MinION is an exciting application of this technology, as the long reads generated allow repeat regions or complex sequences, which cannot be assembled using short read technologies, to be resolved. However, in order to achieve high consensus accuracy, many reads from a single organism are required to provide high coverage and therefore mitigate the error rate of the technology. Additionally, contaminating sequences from other organisms must be present in only negligible quantities. In our hands, it was not possible to predict run output simply based upon the quantity of DNA input into the library preparation. Also important in this regard was our finding of read misassignment between barcode pairs used in the experiment. For limited quantity and high consequence samples, or those in which little is known and therefore purity is an issue, a full flowcell must be considered in order to ensure high quality data is produced. Additionally, the sample could be sequenced with MinION in combination with data generated from short read sequencing in order to accurately call SNPs and for indel analysis. For organisms in which additional non-genomic DNA (ie. plasmids) may be present, careful consideration of *de novo* assemblies is required to mitigate the effect of contaminant sequencing (by a concurrently sequenced organism, or environmental species) on interpretation. In our data, among runs in which high quality data was generated, coverage of the *de novo* assembled genome of the organism of interest was high, whereas that of contaminants was low. Nonetheless, care must be taken in evaluating whether such low coverage contaminant contigs can in fact be differentiated from low copy plasmids which are actually present in a sample.

An alternative strategy proposed by ONT to increase throughput, is to stack samples temporally, possibly storing the flowcell between runs. Of note, our data, in keeping with the findings of others^5,23^, demonstrated that there is both substantial degradation of the flowcell following washing as well as carryover of DNA from the previous run. Thus any stacked runs require additional application of barcoding, and therefore have similar limitations to those described above. This approach may prove useful when for example, an especially important sample is sequenced first, followed later by one of lower value, in order to maximize the sequencing potential of each flowcell.

In an attempt to determine whether, going forward, 1D filtering should be performed in order to improve upon data quality, we evaluated the effect of filter parameters on output. Interestingly, in these datasets, applying a threshold for inclusion did not seem to lead to direct benefits to either *de novo* sequencing output, or to sequence error rates, and had a limited, although beneficial effect on pathogen detection. Also of note is the observation that contaminating sequence data from co-run samples is not filtered out by application of even our most stringent filtering criteria (length >1000, quality >20). This demonstrates that the erroneously barcoded reads were of high quality, and therefore not susceptible to removal through standard filtering practices, and that unless the quantity of mixed reads between samples is sufficiently low, concerns of erroneously attributing small contigs to one sample in mixtures, are well founded. Potentially contaminating reads must, therefore, be addressed either through taxonomic-based filtering post-hoc when distantly related organisms are concurrently sequenced, or by running only a single sample at a time on a flowcell as described above.

The work described in this study is meant to provide new information on the state of ONTs MinION device for whole genome sequencing of bacterial organisms and for assessing its capabilities for pathogen detection in mixed samples. While there are clearly substantial benefits to this technology both for research and clinical applications, several drawbacks including the high error rate, frequent modification of kits and reagents and high levels of interrun-variability, at this time limit the utility of the platform for wide-scale adoption and application. Yet the drastic improvements in data output even through the short time of this study - if indicative of the continued evolutionary trajectory of the technology - will likely further the paradigm shift in the field of long sequencing read analyses. As the development of this technology begins to stabilize, a greater understanding of the benefits and limitations will evolve, with comparisons between experiments and more accurate estimates of sequencing output becoming possible.

## Methods

MinION analyses were carried out independently at two independent laboratory sections at the Public Health Agency of Canada (PHAC), at NML in Winnipeg, Canada. Field-based analyses were conducted in a mobile laboratory stationed at a secure location in Canada. Both labs were part of the MinION early access program, and obtained supplies and reagents between September 2016 and March 2017. Laboratory 1 (L1) performed analyses on a pair of well characterized reference genomes *Yersinia rohdei* (DSM 18270) and *Francisella hispaniensis* (DSM 22475), as well as the “unknown” samples which were part of a remote sequencing exercise. Culture and extraction conditions are described in Supplementary methods. These organisms had been previously sequenced in house, and therefore had Illumina MiSeq (Illumina, Madison, WI, USA) data available as well as closed, high quality reference genomes described in NCBI. Samples analyzed by the second laboratory (L2) were less well characterized *Enterobacter cloacae* and *Escherichia coli* isolates. They were known to harbor plasmid based anti-microbial resistance genes, and were used as a proof of concept for the ability of the MinION to detect and assemble plasmids of importance for antimicrobial resistance (AMR). Sequencing methods tested included replicate 2D sequencing of consecutively added samples, 2D sequencing of both samples simultaneously with the PCR and native barcoding kits, simultaneous 1D sequencing of samples using native barcoding, and Rapid 1D sequencing of single samples (Supplementary Table 1).

### MinION Library Preparation and Sequencing

Upon receipt of flowcells and again immediately prior to sequencing, flowcell pore counts were measured using the Platform QC script (MinKNOW, Supplementary Table 1). Flowcells were replaced into their packaging, sealed with parafilm and tape, and stored at 4 °C until use. Library preparation kits and flowcells used for each experiment are described in Supplementary Table 1. All library preparations were conducted as per the protocols provided by ONT with the exception of the end-prep step where samples were incubated for 10 minutes at both 20 °C and 65 °C. The amount of initial DNA used for both barcoding kits was greater than 100 ng (with the exception of L1- 2D-FAB47257-NAT). Isolate DNA was sheared using Covaris g-tubes (D-Mark Biosystems, Woburn, USA) by centrifuging twice at 4200 rpm for 1 min. Specific conditions for the PCR barcoding kit are described in Supplementary Methods. Purified product for L1 and L2 analyses, was eluted in 30 μl with water. For barcoded libraries, equal quantities of each organism were input to the final library. Completed libraries were loaded onto R9.4 flowcells as per instructions from ONT.

In order to measure the effect of adding sequential samples after initiation of the run, stacked runs were performed, with DNA from a second organism added after the sequencing run had been allowed to proceed for a period of time (4 or 8 hours). In addition the effect of applying a wash step prior to the addition of the second sample was also evaluated in a side-by-side comparison. Given the rapid advancement of the technology, and short time between version releases, data was generated for each run using the most up-to-date methods and protocols available at the time of sequencing. The Mk1B MinION device was used for data acquisition.

#### Laboratory Analysis

Several software tools available through ONT, its subsidiary Metrichor, or open source were used to evaluate the sequence data generated from the MinION runs described in this report. Raw sequence reads were either uploaded to the Epi2Me interface (Metrichor, Oxford, UK), a platform for cloud-based analysis of MinION data which was used for basecalling and demultiplexing, or were processed through Albacore (Oxford Nanopore Technologies, Oxford, UK) on a server at the NML (Supplementary Table 1), or basecalled in real time via MinKNOW. Basic metrics of sequencing abundance and quality were examined for each run using Epi2me when possible. Basecalled data passing Epi2Me quality parameters (q_mean_ > 6) were downloaded off the cloud in fast5 format and converted to fastq or fasta using Poretools (0.6.0)^24^. Only reads designated as pass were included in further analyses. 2D samples which were base called using Albacore/MinKNOW with or without the demultiplexing functionality (v > 1.1.2), had fastq files generated via this program instead. Albacore does not (currently) apply any quality filtering, as such, for repeat native barcoding, all reads which were successfully generated were included in downstream analysis. For a replicated PCR barcoding run, which could not be demultiplexed successfully using Albacore (due to primer incompatibilities with the software), Epi2me was used following base calling for demultiplexing, with no additional filtering applied on output data. Quality was assessed using NanoOK^25^ (v 0.95; 1.22) with read alignment performed using the LAST-align algorithm^26^ (v809). Reference genomes used were obtained from NCBI (*F. hispaniensis* – FSC454, NZ CP018093.1;*Y. rohdei* – YRA, NZ CP009787.1) or from *de novo* assembly of read data with Canu^27^, and polishing of the output contigs using Pilon^8^, in cases in which no high quality genomic references were available.

*De novo* assembly was conducted using Canu for runs in which individual sequence coverage was greater than 20x^27^ (1.3) with contigs polished using Pilon (1.22) and nanopolish (0.8.5) to evaluate optimized assembly quality^8,9^. The 1D run generated by L1 produced an excess of data, and led to a substantial increase in the time taken to perform the assembly using default or customized parameters in Canu. As such a more rapid A-bruijn based approach (Flye 2.3.2)^9^ was also evaluated. In order to determine a useful threshold sequencing requirement for 1D reads, we randomly subsampled sequencing reads obtained from this run, using a customized bash script, and attempted to re-assemble each of the resulting datasets with canu. This dataset was also used to determine thresholds for read quality at varying coverage levels, by assessing their effect on *de novo* assembly quality using Seqtk (trimfq)^28^, porechop and Japsa (jsa.np.filter)^29^. Assemblies were compared to reference genomes using dnadiff^30^.

All software was applied with default parameters, as fine tuning of pipeline protocols was beyond the scope of this manuscript. Where appropriate, investigation of important quality metrics were statistically analyzed and visualized using R (v. 3.4.0).

#### Field-Based analysis

Fast5 files which were classified as pass by the MinKNOW software were subsequently processed and analyzed. The Japsa package^29^ was used to extract fastq reads from fast5 files (npreader), and to filter reads to a minimum average quality threshold of eight (jsa.np.filter). Sequencing reads meeting established criteria were then taxonomically classified using kraken, with default parameters^31^, and a custom database containing various biothreat agents, common environmental and commensal bacterial and viruses, as well as human and ricin genomic DNA as references (database size 21.6 GB) (Supplementary Table 4). This database was constructed with genomes obtained from the NCBI refseq collection on January 9, 2017. As a further confirmatory test for unknown samples, sequences were mapped against a set of signature sequences specific to *B. anthracis* (seven genomic signatures plus the pXO1 and pXO2 plasmids), developed internally, using bwa mem (Supplementary Methods). The first sample was also run through MASH as a method of further confirming the organisms’ identity^11^. At this time, automated scripts were not used, however, analyses of data obtained at the one hour time point were carried out less than 30 minutes following transfer of data.

The datasets generated during the current study are available in the SRA repository, under bioproject PRJNA454306.

The views and opinions expressed herein are those of the authors only, and do not represent the views and opinions of the Public Health Agency of Canada or the Government of Canada.

## Electronic supplementary material

Supplementary Methods and Results

Supplementary Table 3

Supplementary Table 4
