# Supplementary material for: Evaluation of Oxford Nanopore’s MinION Sequencing Device for Microbial Whole Genome Sequencing Applications
Source: Sci Rep. 2018 Jul 19;8:10931. doi: 10.1038/s41598-018-29334-5 (PMC6053456; doi:10.1038/s41598-018-29334-5)
Supplement: Supplementary file 1 — Supplementary Methods and Results [file 41598_2018_29334_MOESM1_ESM.docx]

Evaluation of Oxford Nanopore’s MinION Sequencing Device for Microbial Whole Genome Sequencing Applications

Authors: Andrea D. Tyler^1^, Laura Mataseje^1^, Chantel J. Urfano^1^, Lisa Schmidt^1^, Kym S. Antonation^1^, Michael R. Mulvey^1^, Cindi R. Corbett^1,2^

**Supplementary Information**

**Supplementary Methods**

*Bacterial Culture and DNA extraction*

*Y. rohdei* and *F. hispaniensis* were grown for five days 37°C on blood agar while *E. cloacae* and *E. coli* were grown overnight at 37°C on Luria broth (LB). Cultures then underwent DNA extraction using the MasterPure Complete DNA & RNA Purification kit (Epicenter Illumina, Wisconsin USA) with elution carried out to a final volume of 150 µL in Tris (no EDTA). DNA concentration and purity were evaluated prior to sequencing using the Qubit (Qubit Systems, Ontario, Canada) and Nanodrop 2000 (Thermo Scientific, Massachusetts, USA).

Field tested, unknown samples were prepared as follows: Sample one was composed of *B. anthracis* Vollum strain genomic DNA (1.3 ng/uL), with DNA extraction performed using the MasterPure Complete DNA & RNA Purification kit (Epicenter Illumina, Wisconsin USA), with DNA eluted in TE buffer. Sample two was composed of a swab in TE, containing a mix of 500 uL of *B. anthracis* DNA (0.013ng/uL) and 500 uL of control human genomic DNA (2 ug/uL) obtained from Sigma-Aldrich (Roche, Oakville, Canada).

*MinION Library Preparation and Sequencing*

Upon receipt of flowcells and again immediately prior to sequencing, pore counts were measured using the Platform QC script (MinKNOW various versions). Flowcells were replaced into their packaging, sealed with parafilm and tape, and stored at 4°C until use. Library preparation kits and flowcells used for each experiment are described in Supplementary Table 1. All library preparations were conducted as per the protocols provided by ONT with the exception of the end-prep step where samples were incubated for 10 minutes at both 20°C and 65°C. The amount of initial DNA used for both barcoding kits was greater than 100 ng (with the exception of L1- 2D-FAB47257-NAT). Isolate DNA was sheared using Covaris g-tubes (D-Mark Biosystems, Woburn, USA) by centrifuging twice at 4200rpm for 1min. Specific conditions for the PCR barcoding kit based on expected fragment sizes included: Initial denaturation at 95°C for 3 minutes then 15 cycles (18 cycles L1) of 95°C for 15 seconds, 62°C for 15 seconds and 65°C for 6 minutes; followed by a final extension at 65°C for 10 minutes. PCR purification was performed using 60 μL room temperature AMPure XP beads (Beckman Coulter). Beads were incubated for 5 minutes with rotation followed by two wash steps with 70% ethanol. Excess ethanol was removed and beads were dried briefly. Purified product was eluted in 30 μl with water. For barcoded libraries, equal quantities of each organism were input to the final library. Completed libraries were loaded onto R9.4 flowcells as per instructions from ONT.

In order to measure the effect of adding additional samples after initiation of the run as opposed to using barcoding, stacked runs were performed, with DNA from a second organism added after the sequencing run had been allowed to proceed for a period of time (4 or 8 hours). In addition the effect of applying a wash step prior to the addition of the second sample was also evaluated in a side-by-side comparison. Given the rapid advancement of the technology, and short time between version releases, data was generated for each run using the most up-to-date methods and protocols available at the time of sequencing. The Mk1B MinION device was used for data acquisition.

Field-Based analysis

Fast5 files which were classified as pass by the MinKNOW software were subsequently processed and analyzed. The Japsa package^26^ was used to extract fastq reads from fast5 files (npreader), and to filter reads to a minimum average quality threshold of eight (jsa.np.filter). Sequencing reads meeting established criteria were then taxonomically classified using kraken, using default parameters^27^, with a custom database containing various biothreat agents, common environmental and commensal bacterial and viruses, as well as human and ricin genomic DNA as references (database size 21.6 GB) (Supplementary Table 4). This database was constructed with genomes obtained from the NCBI refseq collection on January 9, 2017. As a further confirmatory test for unknown samples, sequences were mapped against a set of signature sequences specific to *B. anthracis* (seven genomic signatures plus the pXO1 and pXO2 plasmids)*,* developed internally using bwa mem. These signatures are designed to differentiate between this organism and the rest of the *B. cereus* complex group. Positive mapping for a minimum of two of these markers in conjunction with pXO1 and pXO2 has been shown to have a high sensitivity and specificity for *B. anthracis* in a sample (unpublished). Mapping was conducted using bwa mem with the -x 2dont option^28^. While no formal evaluation of mapping parameters was conducted, application of a minimum mapping score threshold (-T) of 50 was tested in order to mitigate the relatively low quality of reads generated through MinION sequencing, alongside evaluation of mapping without this parameter. The first sample was also run through MASH as a method of further confirming the organisms’ identity^29^. At this time, automated scripts were not used, however, analyses of data obtained at the one hour time point were carried out less than 30 minutes following transfer of data.

Supplementary Table 1: Sequencing run protocols and parameters applied for sequencing data obtained in this experiment. For runs in which sequenced organism libraries were not started concurrently, amount of added DNA is described for each. For the barcoded runs, approximately equal amounts of DNA were added for each organism, to the total amount described. FH – Francisella hispaniensis. YR – Yersinia rohdei. BA – Bacillus anthracis. HS – Homo sapien. * Two libraries (generated using the same sample and method) were added to this sequencing run, approximately 1 hour apart.

| Run | Date Run | Input DNA quantity (ng) | 1D/2D | Sequencing protocol | Barcoding | MinKNOW version | Metrichor version | Albacore version |
| --- | --- | --- | --- | --- | --- | --- | --- | --- |
| Laboratory Benchmarking Runs | | | | | | | | |
| L1-2D- FAB29783 | October 6, 2016 | FH-118  YR-128.5 | 2D | SQK-LSK208 | None | Windows: 1.1.17 | 2.42.2 | NA |
| L1-2D-FAB29623 | October 7, 2016 | FH-147.5  YR-103 | 2D | SQK-LSK208 | None | Windows: 1.1.17 | 2.42.2 | NA |
| L1-2D-FAB37836-PCR | December 19, 2016 | 237.5 | 2D | SQK-LSK208/EXP-PBC001 | PCR | Windows: 1.1.21 | Windows: 1.125 (FLO-MIN106) | NA |
| L1- 2D-FAB47257-NAT | Jan 24, 2017 | 72.5 | 2D | SQK-LSK208/EXP-NBD002 | NAT | 1.3.24 | Windows: 2.43.1 (FLO-MIN106) | NA |
| L2- 2D-FAB48048-NAT | Dec 20, 2016 | 106 | 2D | SQK-LSK208/EXP-NBD002 | NAT | 1.1.21 | 1.125 (FLO-MIN106) | NA |
| L2- 2D-FAB47410-PCR | Jan 27, 2017 | 288 | 2D | SQK-LSK208/EXP-PBC001 | PCR | 1.3.25 | 1.125 (FLO-MIN106) | NA |
| L1-2D-FAF14070-PCR | Mar 20, 2017 | 204.5 | 2D | SQK-LSK208/EXP-PBC001 | PCR | 1.5.12 | Linux: 2.45.4 (2D Barcoding) | 1.1.2 |
| L1-2D-FAF12862-NAT | Apr 4, 2017 | 310 | 2D | SQK-LSK208/EXP-NBD002 | NAT | 1.5.12 | NA | 1.1.2 |
| L1-2D-FAF18391 | April 13, 2017 | YR-402  FH-113 | 2D | SQK-LSK208 | NA | 1.5.12 | NA | 1.1.2 |
| L1-1D-FAF18512-NAT | May 10, 2017 | 340.5 | 1D | SQK-LSK108/EXP-NBD103 | NAT | 1.5.12 | NA | 1.1.2 |
| L2-1D-FAF14064-NAT | June 12, 2017 | 266 | 1D | SQK-LSK108/EXP-NBD103 | NAT | 1.5.12 | NA | 1.1.2 |
| Runs Assessing Remote Deployment Capabilities | | | | | | | | |
| L1-1D- FAF06136-RAP | February 6, 2017 | FH - 244 | 1D | SQK-RAD002 | NA | 1.2.8 | NA | 0.8.4 |
| L1-1D-FAF05394-RAP | February 9, 2017 | YR - 184 | 1D | SQK-RAD002 | NA | 1.2.8 | NA | 0.8.4 |
| Remote-FAF12790 – Sample1 | March 8, 2017 | BA - 9.8 | 1D | SQK-RAD002 | NA | 1.3.30 | NA |  |
| Remote-FAF12773 – Sample2 | March 8, 2017 | 0.001 (BA); 75 (HS)* | 1D | SQK-RAD002 | NA | 1.3.30 | NA |  |

Supplementary Table 2: Porecounts for flowcells used during each run described in this report.

| Date | Flowcell | porecount |
| --- | --- | --- |
| Laboratory Benchmarking Runs | | |
| 6-Oct-16 | L1-2D- FAB29783 | 593 |
| 7-Oct-16 | L1-2D-FAB29623 | 1092 |
| 19-Dec-16 | L1-2D-FAB37836-PCR | 1496 |
| 20-Dec-16 | L2- 2D-FAB48048-NAT | 1429 |
| 24-Jan-17 | L1- 2D-FAB47257-NAT | 1091 |
| 27-Jan-17 | L2- 2D-FAB47410-PCR | 989 |
| 20-Mar-17 | L1-2D-FAF14070-PCR | 1134 |
| 4-Apr-17 | L1-2D-FAF12862-NAT | 1155 |
| 13-Apr-17 | L1-2D-FAF18391 | 949 |
| 10-May-17 | L1-1D-FAF18512-NAT | 1400 |
| 12-Jun-17 | L2-1D-FAF14064-NAT | 1275 |
| Runs Assessing of Remote Deployment Capabilities | | |
| 6-Feb-2017 | L1-1D- FAF06136-RAP | 1126 |
| 9-Feb-2017 | L1-1D-FAF05394-RAP | 845 |
| 8-Mar-2017 | Remote-FAF12790 – Sample1 | 1379 |
| 8-Mar-2017 | Remote-FAF12773 – Sample2 | 1458 |

Supplementary Table 3: Assembly statistics in subsampling analysis carried out on L1-1D-FAF18512-NAT. (see excel spreadsheet)

Supplementary Table 4: identity (taxon ID and genbank ID) of organisms included in kraken database used for remote evaluation of MinION reads.

Supplementary Table 5: Basecalling performed using Albacore differed from that carried out via Epi2Me in that no quality filtering parameters were applied following basecalling of reads. Read quality statistics are described for those which were successfully demultiplexed by the software by lab 1. *the quality of data from this run is reported for all sequences which were basecalled with quality >0, as no barcoding was used.

| Run | Mean q | Range | Number >=9 |
| --- | --- | --- | --- |
| L1-2D-FAF14070-PCR | 14.0 | 3.3-24.4 | 5213 |
| L1-2D-FAF12862-NAT | 16.7 | 3.7-24.9 | 3254 |
| L1-2D-FAF18391* | 15.1 | 1-26.7 | 97147 |
| L1-1D-FAF18512-NAT | 11.3 | 4.5-16.0 | 567484 |

Supplementary Figure 1: A) L2 PCR barcoding kmer content plot, generated through nanook. B) Equivalent plot generated by L1 PCR barcoding.


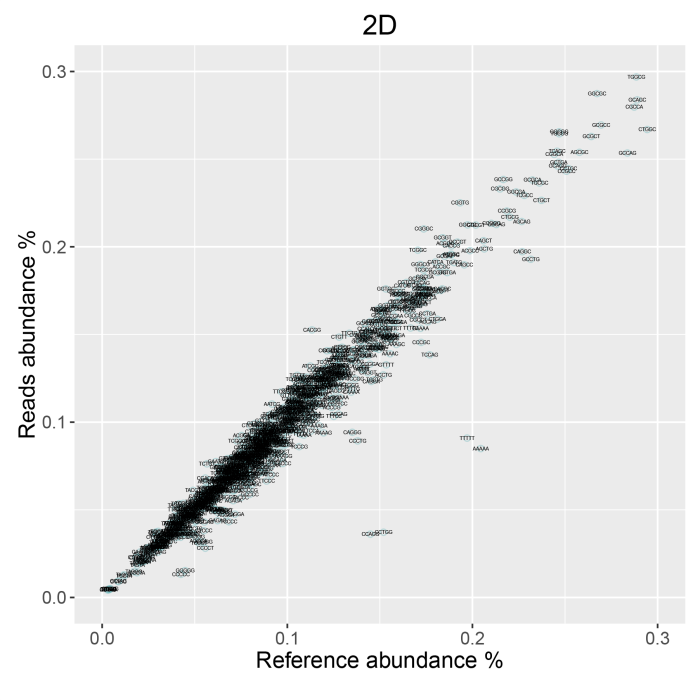

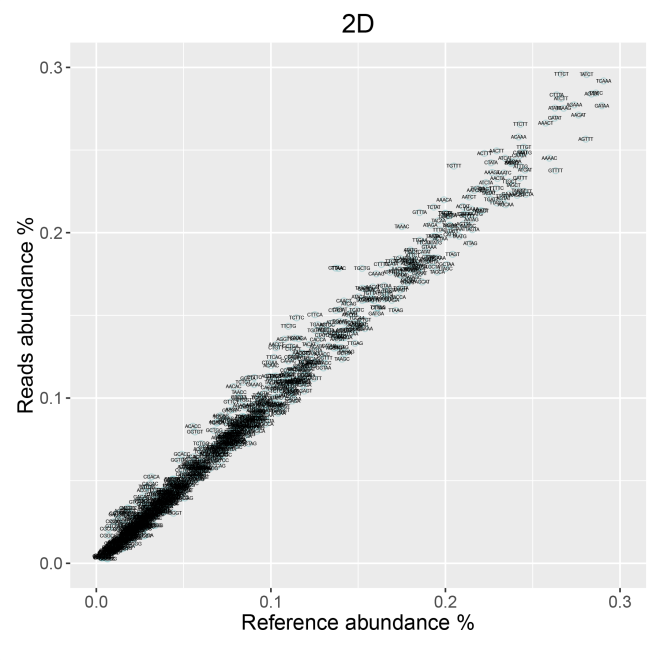


B

A
